# A new species of the genus *Microtendipes* Kieffer, 1915 (Diptera, Chironomidae) from Oriental China

**DOI:** 10.3897/zookeys.212.3329

**Published:** 2012-07-30

**Authors:** Xin Qi, Xiaolong Lin, Xinhua Wang

**Affiliations:** 1 College of Life Science, Taizhou University, Taizhou, Zhejiang 318000, China; 2 College of Life Science, Nankai University, Tianjin 300071, China

**Keywords:** *Microtendipes*, new species, key, catalogue, Oriental Region

## Abstract

A new species of the genus *Microtendipes* Kieffer, 1915, *Microtendipes zhejiangensis* **sp.n.**, is described, and its morphological description and illustrations are given. A catalogue of the genus in Oriental Region is provided and a key to the males of *Microtendipes* in the Oriental Region is given.

## Introduction

*Microtendipes* Kieffer, 1915 is a cosmopolitan genus, occurring in all zoogeographical regions. Immature stagesof *Microtendipes* are found in littoral and sublittoral sediments of large water bodies, with a few species occurring in running water (Ashe et al. 1987; Cranston et al. 1989). So far, there are 61 species recorded around the word.

The Oriental Region includes all of Asia south and east of the Himalayan Mountains (India and South East Asia), as well as southern China and the Islands of Southwestern Japan, Indonesia and Philippines. There was no catalogue of *Microtendipes* for the Oriental Region before this work, some previous records of Oriental *Microtendipes* are as follows: Kieffer (1921) recorded two new species based on the females, *Microtendipes stictopterus* Kieffer, 1921 from Philippines and *Microtendipes dimidiatus* Kieffer, 1921 from Taiwan Province (China), but Edwards (1929a) reviewed the Chironomidae from Philippines and recorded that the type specimen of *Microtendipes stictopterus* in Kieffer (1921) was too damaged for determination, *Microtendipes stictopterus* has been treated as nomen nudum; Reiss (1997) recorded *Microtendipes schuecki* Reiss, 1997 from Thailand; Chaudhuri et al. (2001) listed *Microtendipes callicomus* (Kieffer, 1911) from the Indian subcontinent, but *Microtendipes callicomus* ought to be treated as *Chironomus callicomus*; Wang (2000), Qi and Wang (2006) and Qi and Wang (2010) recorded 8 species of *Microtendipes* from Oriental China; Sasa and Suzuki (2000) recorded *Microtendipes iriocedeus* Sasa & Suzuki, 2000 from Southwestern Japan (Iriomote Island); Kikuchi and Sasa (1990) recorded *Microtendipes tobaquintus* Kikuchi & Sasa, 1990 from Indonesia (Toba Lake).

**Figure 1.**
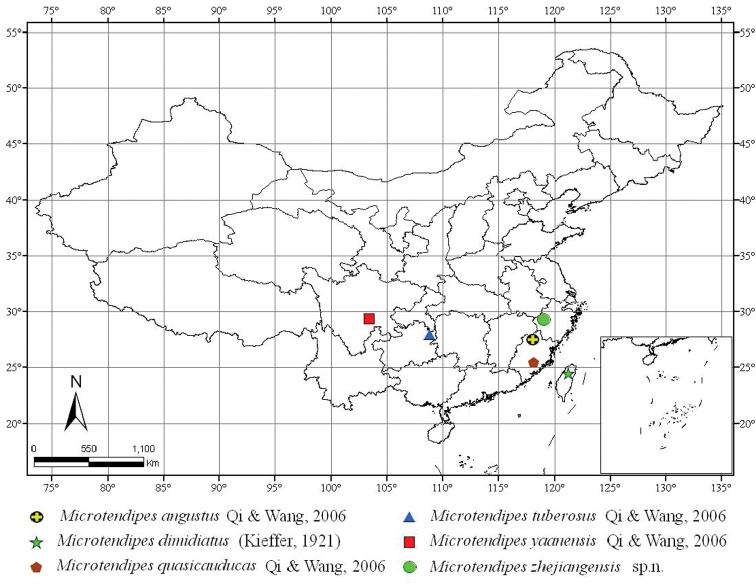
The type localities map of the genus *Microtendipes* in Oriental China.

In this contribution, a new species of *Microtendipes* from Oriental China is described; the type localities map of the genus *Microtendipes* in Oriental China is given (Fig. 1); a catalogue and a key to the species of *Microtendipes* from the Oriental Region are presented.

## Materials and methods

The morphological nomenclature follows Sæther (1980) and the abbreviations of parts measured follow Qi et al. (2012). The material examined was mounted on slides, following the procedure outlined by Sæther (1969). Specimens are deposited in the College of Life Science, Nankai University, China and College of Life Science, Taizhou University, China.

## Taxonomy

### Genus Microtendipes

Kieffer, 1915

http://species-id.net/wiki/Microtendipes

Microtendipes Kieffer, 1915: 70; Pinder 1978: 128; Qi and Wang 2006: 37.

#### Type species.

*Tendipes abbreviatus* Kieffer, 1913 [= *Chironomus chloris* (Meigen, 1818)]

#### Diagnosis.

Most males of *Microtendipes* can be distinguished from all other Chironomini by one or two rows of stout, proximally directed setae on the fore femur. Additionally, the hypopygium of some species generally has a tubercle or wart-shaped median volsella often bearing a tuft of long setae. Species without the above characters require association with immature stages for correct placement in the genus; moreover, *Microtendipes* can be divisible into two species-groups (*pedellus* group and *rydalensis* group) with recourse to immature stages. The characters of larva are as follows: the body is large, red to orange coloured, up to 15 mm long; the antenna has 6 segments; the lauterborn organs alternate on apices of segments 2 and 3; the mandible has 3 inner teeth; the median trifid is either pale or as dark as remaining teeth with very small median tooth (maybe absent); the lateral and ventral tubules are absent.

#### Distribution.

Palaearctic, Oriental, Nearctic, Neotropical and Australian regions.

#### A catalogue of *Microtendipes* in Oriental Region

***Microtendipes angustus* Qi & Wang, 2006**

*Microtendipes angustus* Qi & Wang, 2006: 38.

Oriental China (Fujian, Guizhou Province).

***Microtendipes britteni* (Edwards, 1929)**

*Chironomus (Microtendipes) britteni* Edwards, 1929b: 399.

*Microtendipes britteni* (Edwards): Pinder 1978: 128; Wang 2000: 645; Qi and Wang 2006: 40; Qi and Wang 2010: 497.

Oriental China (Guangdong, Guizhou, Zhejiang Province)

***Microtendipes chloris* (Meigen, 1818)**

*Chironomus chloris* Meigen, 1818: 28

*Microtendipes chloris* (Meigen): Pinder 1978: 128; Qi and Wang 2010: 497.

Oriental China (Zhejiang Province)

***Microtendipes dimidiatus* (Kieffer, 1921)**

*Chironomus (Microtendipes) dimidiatus* Kieffer, 1921: 581

Oriental China (Taiwan Province)

***Microtendipes iriocedeus* Sasa & Suzuki, 200*0***

*Microtendipes iriocedeus* Sasa & Suzuki, 2000: 3, 12.

Southwestern Japan (Iriomote Island)

***Microtendipes pedellus* (De Geer, 1776)**

*Tipula pedellus* De Geer, 1776: 378

*Chironomus aberrans* Johannsen, 1905: 221

*Microtendipes pedellus* (De Geer): Edwards 1929b: 397 ; Wang 2000: 645; Qi and Wang 2006: 41.

Oriental China (Guizhou, Zhejiang Province); South India

***Microtendipes quasicauducas* Qi & Wang, 2006**

*Microtendipes quasicauducas* Qi & Wang, 2006: 41.

Oriental China (Fujian Province)

***Microtendipes schuecki* Reiss, 1997**

*Microtendipes schuecki* Reiss, 1997: 271.

Thailand (DoI Inthanon)

***Microtendipes tobaquintus* Kikuchi & Sasa, 199*0***

*Microtendipes tobaquintus* Kikuchi & Sasa, 1990: 301.

Indonesia (Toba Lake)

***Microtendipes truncatus* Kawai & Sasa, 1985**

*Microtendipes truncatus* Kawai & Sasa, 1985: 18; Qi and Wang 2006: 43.

Oriental China (Zhejiang, Fujian, Guizhou, Yunnan Province)

***Microtendipes tuberosus*** **Qi & Wang, 2006**

*Microtendipes tuberosus* Qi & Wang, 2006: 43.

Oriental China (Guangdong, Guizhou, Hainan Province)

***Microtendipes yaanensis* Qi & Wang, 2006**

*Microtendipes yaanensis* Qi & Wang, 2006: 45; Qi and Wang 2010: 497.

Oriental China (Zhejiang, Sichuan Province)

#### Key to males of the genus *Microtendipes* in Oriental regio^n#^

**Table:** 

1	Hypopygium with median volsella	2
–	Hypopygium without median volsella	7
2	Superior volsella with lateral lobe	3
–	Superior volsella without lateral lobe	5
3	Wing with dark markings	*Microtendipes schuecki*
–	Wing transparent, without markings	4
4	Front femur with small tubercle	*Microtendipes tuberosus*
–	Front femur without small tubercle	*Microtendipes yaanensis*
5	Anal point subtriangular	*Microtendipes pedellus*
–	Anal point parallel sided	6
6	Superior volsella hook-like	*Microtendipes chloris*
–	Superior volsella broad, rounded apically	*Microtendipes truncatus*
7	Inferior volsella abruptly narrowed in apical half	8
–	Inferior volsella digitiform	9
8	Anal point apically slightly swollen and rounded; superior volsella with 4 dorsal setae, 2 basal setae	*Microtendipes angustus*
–	Anal point parallel-sided, slender, apex rounded; superior volsella with 7−10 dorsal setae, 4 long basal setae	*Microtendipes zhejiangensis* sp.n.
9	Wing with dark markings	*Microtendipes quasicauducas*
–	Wing transparent, without markings	10
10	Abdominal tergite VIII narrowed at base, as inverted V-shaped	*Microtendipes iriocedeus*
–	Abdominal tergite VIII not narrowed at base	11
11	Anal point parallel-sided, slender, apex rounded	*Microtendipes tobaquintus*
–	Anal point subtriangular, with pointed apex	*Microtendipes britteni*

# The record of *Microtendipes dimidiatus* was only based on female, so the key does not include it.

### Microtendipes zhejiangensis

sp.n.

urn:lsid:zoobank.org:act:96DD36A0-80C8-4175-9F46-33AC31F7E6B4

http://species-id.net/wiki/Microtendipes_zhejiangensis

Figs 2−5

#### Diagnosis.

The male imago can be distinguished from known species of the genus by the following combination of characters: superior volsella hook-like, apex obtuse, with 7−8 dorsal setae and 4 long basal setae; median volsella absent; absence of pigment marks in wing; acrostichals 2−3.

#### Description.

Male imago (n = 3). Total length 5.75−6.05 mm. Wing length 3.38−3.48 mm. Total length/wing length 1.65−1.79. Wing length/length of profemur 2.25−3.71.

Coloration. Head yellow. Thorax greenish yellow with scutum and postnotum brown. Abdomen greenish yellow, Abdominal tergites I−VI pale green, tergites VII−IX and hypopygium brown. Legs: apical 1/3 of fore femur, basal 1/2 of fore tibia and apical 1/10 of tibiae brown; remaining parts greenish yellow.

Head.AR 1.82−2.48. Temporal setae 16−19 including 6−8 inner verticals, 6−10 outer verticals, and 2−3 postorbitals. Clypeus with 37−38 setae. Tentorium 205−240 mm long, 55−70 mm wide. Palpomere lengths (in mm): 60−65, 63−73, 310−330, 330−350, 460−480. L: 5^th^/3^rd^ 1.39−1.54.

**Figures 2–5.**
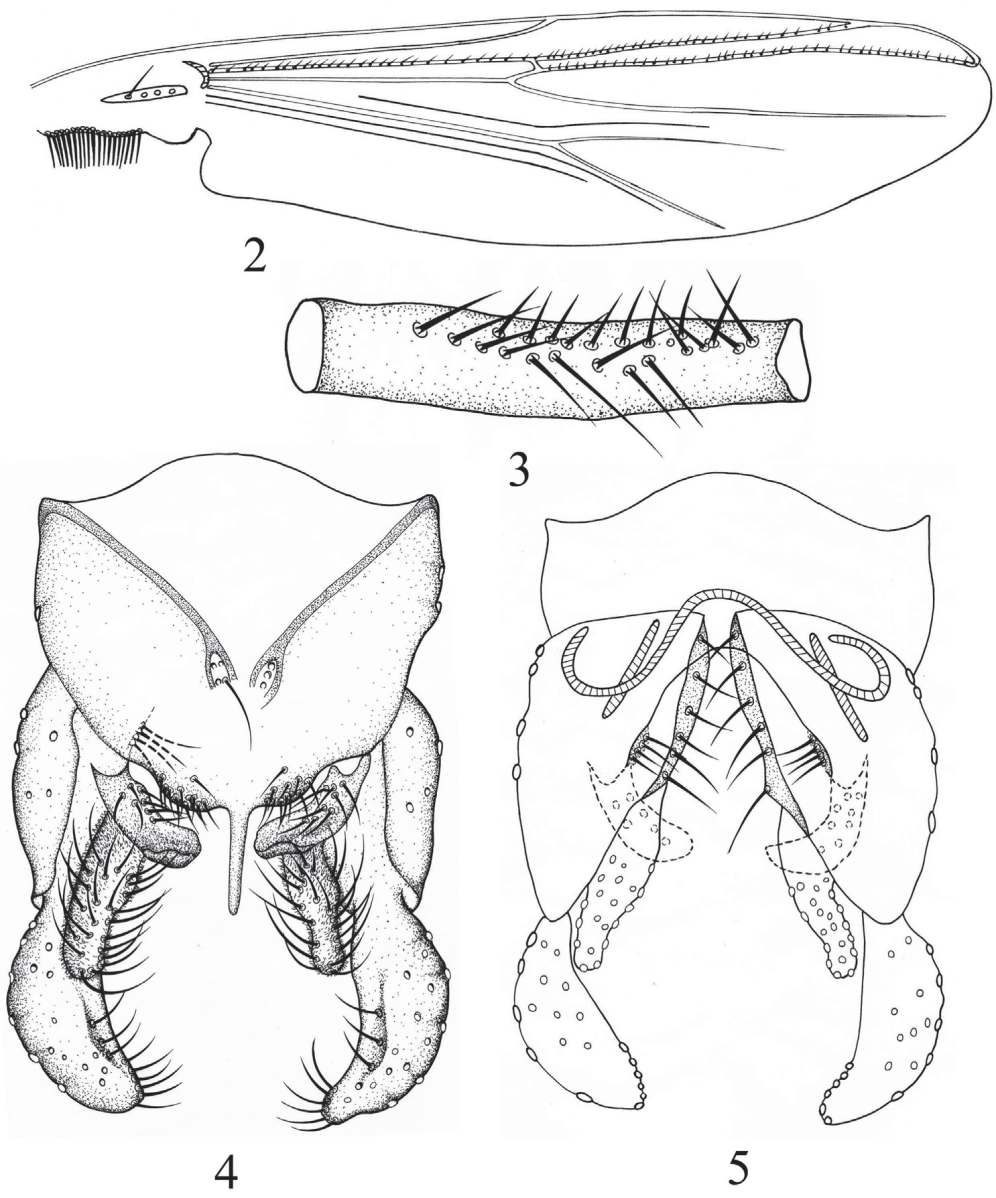
*Microtendipes zhejiangensis* sp.n., male. **2** wing **3** two rows of directed setae in front femur **4** hypopygium (dorsal view) **5** hypopygium (ventral view).

Wing(Fig. 2). VR 1.07−1.13. B with 4 setae; R with 27−28, R_1_ with 24−32, R_4+5_ with 31−49 setae. Squama with 18−20 setae.

Thorax. Dorsocentrals 17, acrostichals 2−3, prealars 4. Scutellum with 10−25 setae.

Legs(Fig. 3). Distal half of front femur with 23−25 proximally directed setae in 2 rows, 180−200 mm long. Spur on median tibiae 25−33 mm long including 28−30 mm long comb, unspurred comb 25−33 mm long; spur on posterior tibia 33−40 mm long including 25−33 mm long comb, unspurred comb 28−30 mm long. Width at apex of front tibia 80−87 mm, of middle tibia 83−85 mm, of hind tibia 90−95 mm. Lengths (in mm) and proportions of legs in Table 1.

Hypopygium(Figs 4−5). Anal point 78−90 mm long, parallel-sided, slender, apex rounded. Tergite IX with 6−9 long setae medially and 22−36 setae along posterior margin. Phallapodeme 90−113 mm long; transverse sternapodeme 50−70 mm long. Gonocoxite 223−238 mm long. Superior volsella 105−125 mm long, hook-like, apex obtuse, with 7−10 dorsal setae and 4 long basal setae. Inferior volsella digitiform, 110−140 mm long, narrowed in apical 1/2, with 29−32 setae. Gonostylus 143−148 mm long, with 9−10 setae along inner margin in distal 1/2. HR 1.51−1.64, HV 4.08−4.32.

Female, pupa and larva are unknown.

#### Type materials.

Holotype: 1♂, China, Zhejiang: Kaihua County, Gutian Mountain, 29°14.27’N, 118°07.13’E, 7.iv.2011, Lin XL, sweeping method. Paratype: 2♂♂, same as holotype.

#### Etymology.

The species is named after the type locality, using the Latin suffix *–ensis*, denoting place of origin.

#### Remarks.

The new species is similar to *Microtendipes pedellus* (De Geer), but can be separated from *Microtendipes pedellus* (De Geer) on the basis of the following: (1) presence of median volsella in *Microtendipes pedellus* (De Geer), with 3 long setae in the median volsella; whereas absence of median volsellain *Microtendipes zhejiangensis* sp.n.; (2) the inferior volsella of *Microtendipes pedellus* (De Geer) digitiform, with 20 setae, whereas the inferior volsella of *Microtendipes zhejiangensis* sp.n. narrowed in apical 1/2, with 29−32 setae; (3) HV of *Microtendipes pedellus* (De Geer) 2.98−3.36, whereas HV of *Microtendipes zhejiangensis* sp.n. 4.08−4.32.

The new species is also similar to *Microtendipes nitidus* (Meigen, 1818). The superior volsella of *Microtendipes nitidus* (Meigen) has a basal expansion bearing more than 5 long setae mesally, but the superior volsella of *Microtendipes zhejiangensis* sp.n. is not expanded basally, with 4 long basal setae separated from each other.

This new species can be separated from *Microtendipes quasicaducas* Qi & Wang on the basis of the following characters: (1) the anal point of *Microtendipes quasicaducas* Qi & Wang slender, tapering from base, and apically pointed; whereas the anal point of *Microtendipes zhejiangensis* sp.n. parallel−sided, slender, apex rounded; (2) the wing with pigment marks in *Microtendipes quasicaducas* Qi & Wang, but the wing without marks in *Microtendipes zhejiangensis* sp.n.; (3) the acrostichals 2−3 in *Microtendipes zhejiangensis* sp.n., whereas the acrostichals of *Microtendipes quasicaducas* Qi & Wang lacking.

This new species can also be separated from *Microtendipes zhamensis* Qi & Wang, 2006 on the basis of the following characters: (1) the anal point of *Microtendipes zhamensis* Qi & Wang slender, pointed; whereas the anal point of *Microtendipes zhejiangensis* sp.n. parallel-sided, slender, apex rounded; (2) presence of median volsella in *Microtendipes zhamensis* Qi & Wang, with 2 setae in median volsella; absence of median volsellain *Microtendipes zhejiangensis* sp.n.; (3) the inferior volsella of *Microtendipes zhamensis* Qi & Wang digitiform, with 24 setae; whereas the inferior volsella of *Microtendipes zhejiangensis* sp.n. narrowed in apical 1/2, with 29−32 setae; (4) the acrostichals 2−3 in *Microtendipes zhejiangensis* sp.n., whereas the acrostichals of *Microtendipes zhamensis* Qi & Wang lacking.

#### Distribution.

The species is known from Zhejiang Province of China.

**Table1.** Lengths (in µm) and proportions of legs of *Microtendipes zhejiangensis* sp.n. (n = 3).

	_P_1	_P_2	_P_3
fe	1375−1500	1625−1700	1875−1875
ti	1550−1700	1525−1575	1700−1800
ta_1_	1900−2075	1025−1100	1250−1300
ta_2_	875−975	525−550	750−825
ta_3_	875−950	420−450	575−600
ta_4_	775−825	275−290	350−360
ta_5_	325−350	150−150	150−160
LR	1.21−1.22	0.68−0.72	0.71−0.72
BV	1.70−1.72	3.00−3.04	2.52−2.64
SV	1.54−1.55	2.93−3.05	2.83−2.90
BR	2.21−2.50	3.40−3.75	2.67−4.75

## Supplementary Material

XML Treatment for Microtendipes

XML Treatment for Microtendipes zhejiangensis
